# Self‐Efficacy of General Practitioner Family Physicians in Mental Health Services: A Cross‐Sectional Study in Turkey

**DOI:** 10.1111/jep.70097

**Published:** 2025-04-13

**Authors:** Emel Güden, Arda Borlu, Özlem Olguner Eker, Saliha Özsoy, Zeynep Baykan

**Affiliations:** ^1^ School of Health Sciences Cappadocia University Ürgüp Nevşehir Türkiye; ^2^ Public Health Department, Faculty of Medical Erciyes University Talas Kayseri Türkiye; ^3^ Psychiatry Department Erciyes University Talas Kayseri Türkiye; ^4^ Medical Education Department Erciyes University Talas Kayseri Türkiye

**Keywords:** community health care, competence, general practice, mental health, physicians, primary care, self assessment

## Abstract

**Rationale:**

The role of primary care physicians in mental health services is increasingly significant. However, there is a lack of research on general practitioners' interest and self‐efficacy in providing mental health services.

**Aims and Objectives:**

This study aims to assess the interest and self‐efficacy of general practitioners in mental health services and to identify their educational needs in this area.

**Methods:**

This study employs a cross‐sectional design. A total of 461 family physicians are working in primary care health services in Kayseri, Turkey. The study sample included 415 general practitioners who had not received specialist training in family medicine after graduating from medical school. Face‐to‐face surveys were conducted with 270 general practitioners who agreed to participate in the study. The survey included questions about demographic characteristics, postgraduate training, experiences related to mental health, knowledge of mental health and medication treatment, self‐evaluations of self‐efficacy in mental health services, and requests for education on the subject.

**Results:**

General practitioners reported low self‐efficacy in the use and dosage of psychiatric medications (11.9%), but felt more competent in relation to drugs with addictive potential (34.4%). However, they generally perceived their competence in the use and monitoring of psychiatric medications to be low. The area where they felt least competent in managing mental illnesses in primary care was “intervention in suicide.” Their awareness and coordination regarding community mental health centers, as mental health service providers, were found to be low. Overall, general practitioners perceived themselves as inadequately competent in tracking and managing mental illnesses.

**Conclusions:**

General practitioners acknowledge that mental health services are a primary care responsibility. However, there is a need to increase their self‐efficacy in providing mental health services at the primary care level. Since all participants in this study were public employees, continuing mandatory postgraduate mental health training is crucial. Additionally, strengthening collaboration and coordination mechanisms, as well as providing more effective referrals to community mental health centers, is essential. These efforts will significantly contribute to improving the community's mental health.

## 1 Introduction

In recent years, the prevalence of mental illnesses and psychological disorders has been steadily increasing. According to data from the World Health Organization (WHO), mental disorders and substance use have risen by approximately 13% over the past decade. Globally, around 20% of children and adolescents suffer from a mental disorder, and suicide ranks as the second leading cause of death among individuals aged 15–29. One in ten people (10.7%) live with a mental disorder, corresponding to approximately 792 million individuals [1]. Mental illnesses are among the top 20 health issues leading to disability worldwide, with depression ranking as the third leading cause of disability. By 2030, depression is projected to become the leading cause of disability [2].

The widespread prevalence of mental disorders presents significant challenges, particularly in low‐ and middle‐income countries, where access to mental health services remains inadequate. Due to the shortage of specialized mental health services, the WHO emphasizes the need to enhance the role of primary healthcare services in mental healthcare. Training primary healthcare professionals in mental health care is expected to facilitate early diagnosis and intervention, ultimately improving the referral of psychiatric patients to appropriate healthcare services.

Primary healthcare services play a crucial role in the early diagnosis, follow‐up, and treatment of both physical and mental illnesses. Primary care physicians are at the forefront of managing patients' long‐term health needs, identifying and addressing both physical and psychological conditions. It has been reported that individuals with mental disorders seek primary healthcare services at least once [3]. In this context, primary care physicians are well‐positioned to closely monitor their patients' psychosocial conditions, disease progression, and periodic changes, providing opportunities for early intervention and disease prevention. Moreover, primary healthcare services contribute to reducing the stigma associated with psychiatric disorders, thereby increasing patients' willingness to seek mental health support and adhere to treatment [4].

In Turkey, primary healthcare services are delivered by family physicians. Under the family medicine system, which was introduced in 2005, medical school graduates can be appointed as family physicians, while those who complete specialized training in family medicine qualify as “family medicine specialists.” Although the number of family medicine specialists has been increasing over the years, the majority of family physicians in Turkey are still general practitioners (GP) [5]. Mental health services in Turkey are predominantly provided by specialist psychiatrists. Psychiatric disorders rank second after cardiovascular diseases in terms of disease burden, accounting for 19% of the total burden, thus representing a significant public health issue. However, studies indicate that individuals with mental disorders have a low preference for seeking primary healthcare services [6]. One of the primary reasons for this is the non‐compulsory referral system in Turkey, which allows patients to directly access specialists in public, university, or private hospitals. Another contributing factor is dissatisfaction with primary healthcare services. Although patients frequently visit family physicians to obtain prescriptions for psychiatric medications, their trust in primary healthcare services for mental health issues remains low [7].

Since 2008, the primary institutions responsible for the treatment and follow‐up of diagnosed psychiatric patients in Turkey have been Community Mental Health Centers (CMHCs) [8]. CMHCs provide information to individuals with severe mental illnesses and their families, offer outpatient treatment services, and implement various therapeutic interventions such as rehabilitation, psychoeducation, occupational therapy, and individual and group therapy. These centers aim to improve patients' ability to live independently within the community while collaborating with psychiatric clinics and conducting home visits when necessary. Primary care physicians, who are the first to see patients, establish diagnoses, monitor medication use, and assess other health concerns, play a vital role in this process. Therefore, coordination between family physicians and CMHCs is essential for ensuring the continuous and structured follow‐up of psychiatric patients [9, 10].

Despite the critical role of primary healthcare in the recognition, diagnosis, and management of mental disorders, research on this subject remains insufficient. Existing literature highlights gaps in knowledge regarding psychiatric medication use, patient follow‐up, and primary healthcare professionals' approaches to mental health. Additionally, Turkey has been hosting a significant number of refugees due to war, climate crises, and other humanitarian emergencies, necessitating the provision of healthcare services to these populations. Furthermore, large‐scale natural disasters—such as the 2023 earthquakes—and economic challenges have further underscored the importance of primary healthcare services in addressing mental health needs.

This study aims to assess the mental health knowledge, approaches to managing specific psychiatric disorders, confidence levels in handling mental health cases, and knowledge gaps concerning psychiatric medication among general practitioner family physicians working in primary healthcare settings in Kayseri, Turkey. Additionally, by identifying their educational needs, this study seeks to contribute to the development of health policies aimed at improving primary mental healthcare services.

## 2 Materials and Methods

### 2.1 Study Design

A cross‐sectional study was conducted targeting GPs providing primary healthcare services in Kayseri, Turkey, following the STROBE criteria.

### 2.2 Study Population and Sample

The total number of GPs working in public primary healthcare services in Kayseri province is 461. The ideal sample size, determined with a ±5% margin of error at a 95% confidence level, is 200 individuals. Response rates from similar studies (ranging from 35% to 56%) were used as reference values for sample selection [11, 12].

### 2.3 Gps Included in the Study

To be included in the study, participants must work as GPs in Kayseri and consent to participate in the research. There were no age or gender restrictions in our study. Individuals who have undergone family medicine specialty training have also received training in mental health, unlike GPs. However, they were excluded from the study as their inclusion could potentially affect the results. A total of 191 individuals, consisting of family medicine specialists (*n* = 46) and those who declined to participate in the study (*n* = 145), were excluded from the research. Ultimately, 270 individuals consented to participate in the study by signing the informed consent form, representing a participation rate of 65.1%.

### 2.4 Data Collection Tools

Data were collected using a self‐administered questionnaire designed for GPs. The survey was divided into four sections. The first section included 10 questions about participants' socio‐demographic characteristics and experiences related to mental health problems. The second section consisted of 18 questions measuring participants' perceptions of competence regarding mental illnesses encountered in primary care and the medications used. The third section evaluated participants' level of knowledge about mental illness and pharmacological treatment, consisting of nine questions. Finally, two open‐ended questions were included in the questionnaire. The questionnaire comprised a total of 39 questions in Turkish and could be completed in approximately 10 min.

### 2.5 Socio‐Demographic Characteristics and Experiences With Mental Problems

Information regarding GPs age, gender, marital status, total years of professional experience, workplace location, perception of mental health services as part of their duties, postgraduate mental health training status, history of psychiatric illness, and presence of a family member with a psychiatric illness was collected in this section.

### 2.6 Self‐Assessment of Approach and Management of Mental Disorders

Participants were asked to evaluate their own approaches to each fundamental medical practice. The response options included “I am competent,” “I am not competent,” and “I am undecided.” The items were designed in accordance with the competencies outlined in the National Core [13]. Interclass correlation (ICC) was calculated to assess the test‐retest reliability of the questions. The ICC value was found to be 0.849 with a 95% confidence interval when the questionnaire results of 50 individuals were compared 12 weeks after the initial test.

### 2.7 Thoughts on Mental Illnesses and Pharmacological Treatment

Common mental health problems and pharmacological and nonpharmacological treatments found in WHO and Turkish Ministry of Health training guides were included in our questionnaire. The questions were answered as “True,” “False,” or “I don't know.” Interclass correlation (ICC) was calculated to assess the test‐retest reliability of the questions. The ICC value was found to be 0.884 with a 95% confidence interval when the questionnaire results of 50 individuals were compared eight weeks after the initial test.

### 2.8 Open‐Ended Questions

Two open‐ended questions were asked in this category. Participants were asked to freely list the causes of mental illnesses and the topics they wanted to receive education on mental health. The provided information was coded, with similar content classified under the same title and different content under different titles.

### 2.9 Ethics and Permissions

The research was conducted in accordance with the World Medical Association's Helsinki Declaration. Approval was obtained from the Clinical Research Ethics Committee (138706). Participants were asked to participate in the survey after providing informed consent.

### 2.10 Questionnaire Administration

Survey forms were distributed to participants' workplaces by researchers between September 1 and November 15, 2022, with a 1‐week period given for completion. After one week, the forms were collected again. The distribution and retrieval of questionnaires were carried out through postal service providers of the provincial health directorate.

### 2.11 Statistical Analysis

The data from the study were analyzed using the SPSS 26 software package. Descriptive statistics, including frequencies and percentages, were provided for data evaluation and presented through tables and graphs. The Chi‐square test was used to assess differences between independent variables.

The survey results assessing the self‐evaluation of approaches to and management of mental disorders are presented in the findings section using the response options: “I am competent,” “I am not competent,” and “I am undecided.” For knowledge‐based questions, only the correct responses are reported under the “true or false” options. Open‐ended responses regarding the perceived causes of mental disorders and the topics participants wish to receive further training on were coded, and the obtained data were visualized using graphical representations.

## 3 Findings

A total of 270 GPs agreed to participate in the study (65.1%). The mean age of family physicians was 47.35 ± 8.60 years. The average duration of professional experience was 19.73 ± 9.01 years. Of the participants, 66.3% were male, 81.1% worked in urban family medicine units, and 53.7% had not received any postgraduate mental health training. Additionally, 24.8% of GPs reported experiencing a mental health problem themselves, while 26.3% reported at least one close relative with a mental health problem. Furthermore, 68.5% of GPs stated that primary mental health services were within their job description. The demographic characteristics of the participants are shown in Table 1.

**Table 1:** Demographic characteristics of primary care general practitioners.

n:270	X^2^	SD
**Age**	47.35	8.60
**Professional Year**	19.73	9.01
	**Number**	**%**
**Gender**		
Female	91	33.7
Male	179	66.3
**Marital status**		
Married	241	89.3
Single	29	10.8
**Working place**		
Urban	219	81.1
Rural	51	18.9
**Receiving postgraduate mental health education**		
Received	125	46.3
Not Received	145	53.7
**Having a relative/close friend with a mental illness**		
Have	71	26.3
Have not	199	73.7
**Experienced a mental illness at some time of his/her life**		
Have	67	24.8
Have not	203	75.2
**The belief that offering mental health service is among her/his duty**		
Exactly his/her duty	185	68.5
Partially his/her duty	7	2.6
Not his/her duty	73	27
No idea	5	1.9

### 3.1 Self‐Assessment of Approach and Management of Mental Illness

GPs reported feeling inadequately qualified in the use and dosage of psychiatric medications for pregnant or breastfeeding patients (%11.9). However, they felt more competent regarding “knowing which groups of psychiatric drugs have addictive potential and informing patients about this” (%34.4). Overall, it was found that general practitioners felt their competence in the use and monitoring of psychiatric medications was low (Table 2).

**Table 2:** Primary care physicians’ perceptions regarding their competence in psychiatric knowledge and skills.*

Assessment of my competence about my pharmacological knowledge and skills (*n*:270)	Adequate		Insufficient		Undecided	
	*N*	%	*N*	%	*N*	%
Identifying psychiatric medication groups with addiction potential and informing patients about this issue	93	34,4	92	34,1	85	31,5
Knowing psychiatric medication indications	67	24,8	111	41,1	92	34,1
Knowing the duration of use and treatment completion periods for psychiatric medications	66	24,4	119	44,1	85	31,5
Managing and knowing psychiatric medication use in elderly patients	55	20,4	133	49,3	82	30,4
Knowing the side effects of psychiatric medications	53	19,6	135	50,0	82	30,4
Knowing the contraindications of psychiatric medications	47	17,4	136	50,4	87	32,2
Knowing the interactions between psychiatric medications and other drugs	35	13,0	145	53,7	90	33,3
Knowing the use and dosages of psychiatric medications in pregnant or breastfeeding patients	32	11,9	166	61,5	72	26,7

Assessment of my competence in basic mental health practices	*N*	%	*N*	%	*N*	%
Differentiating between medical and mental illnesses	158	58,5	42	15,6	70	25,9
Taking psychiatric history	149	55,2	60	22,2	61	22,6
Assessing Mental State	144	53,3	55	20,4	71	26,3
To ensure the continuity of follow‐up and treatment of patients recommended by the psychiatrist	106	39,3	67	24,8	97	35,9
Conducting studies on preventive mental health services	103	38,1	70	25,9	97	35,9
Assessing Suicide Risk	59	21,9	102	37,8	109	40,4
Knowing the duties and objectives of CMHC	57	21,1	115	42,6	98	36,3
Knowing which illnesses will be referred to the CMHC	52	19,3	118	43,7	100	37,0
Collaborating with the CMHC for communication and referral	37	13,7	136	50,4	97	35,9
Intervention for suicide	28	10,4	135	50,0	107	39,6

* The items listed in Table 2 were compared based on age, years of professional experience, workplace, gender, history of prior mental health issues, and acquaintance with someone experiencing mental health problems, and the results were found to be nonsignificant (*p* > 0.05).

Regarding the follow‐up and management of mental illnesses in primary care, GPs felt more competent in “distinguishing between medical and mental illnesses and referring patients to mental health professionals” (58.5%). However, the lowest level of competence was observed in the area of “intervening in cases of suicide” (10.4%). Generally, GPs perceived themselves to be inadequately competent in the follow‐up and management of mental illnesses (Table 2).

Primary mental health services are provided by CMHCs, which ensure coordinated delivery of mental health services with trained mental health service providers. Participants reported low levels of competence in referring individuals with mental health problems to CMHCs, communicating with these centers, and knowing which diseases should be referred to these centers (Table 2).

### 3.2 Knowledge

Participants responded to statements about various mental health problems and psychiatric drugs as “true,” “false,” or “I don't know.” The results of those who provided correct answers to the statements are shown in Table 3. The statement with the least correct responses was “One should not ask if someone with depression has thoughts of suicide.”

**Table 3:** The correct answer rates of general practitioners regarding specific mental disorders and psychotropic medications.

Specific Conditions (n:270)	*N*	%
Unexplained physical pain or fatigue may be a symptom of depression	239	88,5
An early diagnosis of mental health disorders can increase the chances of recovery	230	85,2
All psychiatric medications have the potential to cause dependence	211	78,1
Psychiatric medication is not used during pregnancy	186	68,9
An individual can cease hoarding disorder (possessions, etc.) at any time	149	55,2
In psychiatric disorders, herbal products can be used instead of medication	138	51,1
An individual with depression can recover spontaneously without any treatment	138	51,1
Psychiatric medications should only be initiated by psychiatrists	115	42,6
Asking whether someone experiencing depression has suicidal thoughts should be avoided	101	37,4

Volunteers participating in the study reported obtaining information about current psychiatric drugs from university psychiatry professors (45.9%), medication leaflets (40.7%), pharmacology textbooks (38.1%), colleagues (20.0%), the Turkey Drug Guide (18.9%), and promotional events by pharmaceutical companies (18.9%).

Furthermore, 81.5% of the participants reported recommending nonpharmacological methods to individuals showing symptoms of mental illness. Among these, 66.3% mentioned exercise and sports, 61.1% mentioned seeing a psychologist, 47.0% mentioned going on vacation, 41.9% mentioned prayer/worship/yoga/meditation, and 11.1% mentioned herbal methods as beneficial.

**Figure 1:**
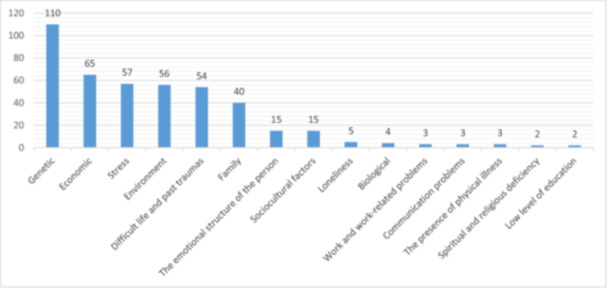
Causes of mental illnesses as perceived by general family practitioners.

Participants' thoughts on factors contributing to mental health problems, as addressed in open‐ended questions, are shown in Figure 1. Genetic factors were identified as the most common cause of mental illness, followed by economic factors, stress, environment, difficult life experiences, and trauma history.

**Figure 2:**
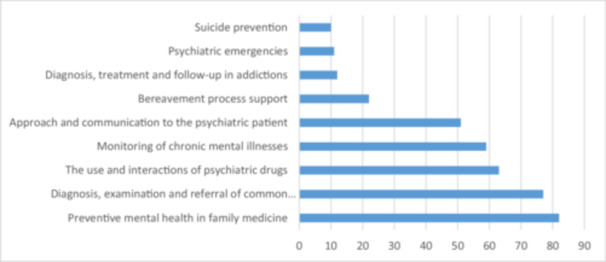
Preferred topics for mental health training among general family practitioners.

Furthermore, participants were asked about the topics they would like to receive education on regarding mental health in open‐ended questions, and the responses received are presented in Figure 2. The most preferred education topics were preventive mental health in family medicine, diagnosis and referral of common mental illnesses, use and interactions of psychiatric drugs, follow‐up of chronic mental illnesses, and communication with mental health patients.

## 4 Discussion

The integration of nonspecialist healthcare personnel into mental health services is an important step in addressing the demand for mental healthcare, particularly in low‐ and middle‐income countries. Primary care mental health services are a crucial area for ensuring that patients receive comprehensive healthcare. However, primary care providers must also be adequately prepared to deliver mental health services. This study examines the knowledge levels of GPs in primary care regarding the management of mental illnesses, the use and monitoring of psychiatric medications, and their self‐assessments of their approach to and management of mental health conditions.

Based on our findings, we can conclude that general practitioners in primary care do not feel sufficiently confident in diagnosing mental disorders, prescribing medications, and referring patients for advanced treatment. The first step in effectively implementing mental health services in primary care is to recognize this responsibility as part of one's professional duties. A significant portion of the general practitioners who participated in our study acknowledged this responsibility. However, more than half of the group had not participated in any mental health training programs after graduation. The literature similarly indicates low levels of training in mental health topics [14, 15]. This could be considered an indicator of low participation in professional development. Additionally, the average of 20 years of professional experience among the general practitioners in our study suggests a considerable gap in acquiring up‐to‐date knowledge. The reported lack of confidence, particularly in the use and monitoring of psychiatric medications, underscores their need for current information in this field.

Among the participants, less than 25% considered themselves adequately knowledgeable about the indications, duration of use, drug interactions, and contraindications of psychiatric medications. In a similar study conducted with primary care physicians in Greece, approximately half of the participants reported having sufficient knowledge of the effects of psychiatric medications [12]. In a cross‐sectional study conducted in Albania, Bulgaria, Moldova, Romania, and Serbia, 40.9% of general practitioners stated that they could treat depression with medication, while 79.4% believed that referring patients in need of antidepressants to a psychiatrist would result in better outcomes [16]. Considering these findings alongside the literature, it becomes evident that there is a need to expand training programs and resources related to psychiatric medications to improve primary care services. Moreover, these findings also highlight the necessity of specialist support to effectively manage mental health issues.

Another area requiring the support of specialist healthcare personnel is the management of psychiatric medications in pregnant and lactating women. The participants in our study demonstrated the lowest proficiency in the use and dosing of psychiatric medications for these patients. This finding is particularly noteworthy as it relates to maternal and child health, a significant component of primary care.

Accurate diagnosis is essential for the effective treatment of mental illnesses. Common psychiatric disorders encountered in primary care often necessitate rapid diagnosis and treatment decisions due to high patient volume. The quick assessment and diagnosis of mental disorders require clinical skills grounded in the knowledge and experience of family physicians. Among the participants in our study, over 50% considered themselves competent in diagnosing mental illnesses. However, a study conducted in Italy reported that the rate of false‐positive diagnoses of depression by primary care physicians was 45%, with 26.9% of these individuals being prescribed antidepressants. This situation was attributed to the inclusive approach that primary care physicians tend to adopt when diagnosing depression [17].

Half of the general practitioners who participated in our study believe that depression can resolve on its own. Depression and anxiety are among the most common mental health issues. It is crucial for primary care physicians to have a more informed understanding of these conditions. These disorders can be treated effectively at the primary care level, and early diagnosis can prevent more severe outcomes, such as major depression and suicide [18].

Among the participants, the statement most frequently answered incorrectly was, “One should not ask someone with depression about suicidal thoughts.” A survey conducted among primary care physicians in Germany reported that 23% of respondents chose to avoid discussing suicide with elderly patients exhibiting symptoms of depression due to concerns about potentially triggering suicidal ideation [19]. The World Health Organization (WHO) has published guidelines on how to approach this subject and has recommended that healthcare professionals receive appropriate training in this area [20]. In our study, only 21.9% of participants described themselves as competent in assessing suicide risk, a proportion that is notably low for frontline healthcare providers. Existing literature indicates that individuals who die by suicide often consult a general practitioner in the months preceding their death [21]. Furthermore, among the fundamental medical practices related to mental health evaluated in our research, “Suicide Intervention” had the lowest self‐reported competency rate at 10.4%. Similar studies have also identified suicide intervention as the area where primary care physicians feel least competent [22]. It has been suggested that healthcare professionals' low self‐perceived competence in managing psychiatric disorders and suicidal patients poses a significant barrier to effective intervention [23]. Notably, the general practitioners participating in our study expressed both interest in and a desire to receive training specifically focused on suicide‐related topics. The participants in our study expressed interest in receiving education on mental health topics related to “suicide.” The necessity for adequate training in this area is highlighted, considering the low level of perceived competence among primary care physicians.

Monitoring mental illnesses in primary care is sometimes insufficient for resolving all issues. Referral to a mental health specialist or a community mental health center may be necessary. It is the primary care physician's responsibility to ensure the appropriate patients are referred to these centers. In our study, 21.1% of general practitioners reported having sufficient knowledge about community mental health centers, while 19.3% considered their knowledge adequate regarding the conditions that warrant referral to these centers. Only 13.7% felt competent in communicating with these centers. These findings highlight the need for developing sustainable solutions to improve coordination between primary care physicians and community mental health centers.

CMHCs are facilities that provide outpatient services to patients with chronic mental health issues within a community‐based framework during weekday working hours. These centers offer familial and social support to patients and contribute to the effective treatment of mental disorders by providing alternative therapeutic methods. The community‐based mental health model implemented in Turkey encompasses primary care (Family Medicine), secondary care (State Hospitals, Mental Health Hospitals), and tertiary care (Universities). Within this model, after being treated in a clinic for a certain period, patients are discharged, and their treatment and care continue within their community. During this process, the patient receives familial and social support, and alternative therapeutic methods are applied [24].

Limitations of this study include the specific association of the participant group with a particular region and institution, limiting the generalizability of the results. The use of a questionnaire for data collection may lead participants to categorize their thoughts. The absence of a proficiency scale for mental health services in primary care reduced data standardization. These limitations should be taken into account for future research and should be addressed more comprehensively.

## 5 Conclusion and Recommendations

The findings of the study indicate that the self‐confidence of GPs in mental health services needs to be enhanced. In this regard:

The knowledge levels of GPs in mental health should be increased. Participation in postgraduation training programs should be encouraged, and access to current information should be facilitated.

More effective communication should be established between primary care providers, mental health specialists, and community mental health centers. The promotion of community mental health centers and the encouragement of GPs to refer patients to these centers more effectively should be prioritized.

The management skills of GPs regarding the assessment of suicide risk and the necessary interventions should be improved.

Continuous improvement processes should be adopted in health policies. It is important to clarify the roles of GPs in mental health services and to share best practices.

In conclusion, enhancing the capacity of GPs in mental health services is essential for the overall health and well‐being of the community. Studies conducted in this direction could lead to improved service delivery for patients and serve as an effective strategy in addressing mental health issues.

## Conflicts of Interest

The authors declare no conflicts of interest.

## Data Availability

The data that support the findings of this study are available on request from the corresponding author. The data are not publicly available due to privacy or ethical restrictions.
